# A Pilot Study of Prediction of Creatinine Clearance by Ellipsoid Volumetry of Kidney Using Noncontrast Computed Tomography

**DOI:** 10.31662/jmaj.2018-0021

**Published:** 2019-02-20

**Authors:** Mitsuhiro Matsuo, Fuminori Yamagishi, Akiko Higuchi

**Affiliations:** 1 Department of Internal Medicine, Itoigawa General Hospital, Itoigawa, Niigata, Japan; 2 Department of Surgery, Itoigawa General Hospital, Itoigawa, Niigata, Japan

**Keywords:** preoperative data, renal parenchymal volume, elderly population

## Abstract

**Introduction:**

Aging is associated with a decline in kidney volume and function. The purpose of this study is to investigate a direct relationship between kidney volume and function in the elderly population and to challenge whether kidney function could be predictable by using the kidney volume.

**Methods:**

We conducted a chart review of 366 patients who underwent abdominal computed tomography (CT) and renal function measurement prior to gastrointestinal surgery. The kidney volume was calculated by the ellipsoid method using a coronal section of noncontrast CT images.

**Results:**

The patients were 72.2 ± 13.2 years of age, and 39.0% were female. Their average measured creatinine clearance (mCCr) was 72.0 ± 21.5 mL/min. The average kidney volume was 100.3 ± 27.6 cm^3^ in the right kidney and 109.3 ± 30.9 cm^3^ in the left. There was a significant positive correlation between the total kidney volume and mCCr. Multivariate regression analysis showed that age, diabetes mellitus, and total kidney volume were dependent variables with which to predict mCCr. The use of total kidney volume predicted mCCr of ≥50 mL/min with moderate accuracy (area under the curve = 0.782; 95% confidence interval = 0.692–0.871).

**Conclusions:**

These results indicate a direct relationship between kidney volume and function in the elderly and might provide a pilot method which estimates the renal function using kidney morphology obtained from pre-existing CT images.

## Introduction

 Aging is well recognized to be associated with a decline in kidney function ^(1)^. Age is one of the most important dependent variables in the Cockcroft–Gault equation ^(2)^ and in formulas for estimating glomerular filtration rate (GFR) ^(3), (4)^. As aging progresses, the kidney also becomes atrophic due to atherosclerosis and/or glomerulosclerosis ^(5)^. Although a positive correlation between the renal parenchymal volume and GFR has been shown among the relatively young population ^(6), (7)^, there are limited data evaluating the direct relationship between kidney function and its volume in the elderly population. Some studies showed a positive relationship between kidney function and morphology in the elderly by using estimated GFR (eGFR) and/or ultrasonography ^(8), (9)^. However, eGFR is just an estimated value, and ultrasonography is less quantitative ^(10)^. The use of computed tomography (CT) to assess kidney size by the ellipsoid method is reportedly more accurate than ultrasonography ^(11)^. We conducted a chart review of patients who underwent abdominal CT and creatinine clearance (CCr) measurement prior to gastrointestinal surgery. These preoperative data were analyzed to clarify the relationship between kidney volume and function.

## Materials and Methods

### Study design

This retrospective, cross-sectional study was approved by the Ethics Committee of Itoigawa General Hospital (No. 2017-9), and conducted in accordance with the principles of the Declaration of Helsinki. A retrospective medical chart review was performed using electronic medical records to identify patients who underwent gastrointestinal surgery from January 2014 to December 2016 at our hospital. Age, sex, body height, body weight, comorbidities, operative procedure, laboratory data, and noncontrast abdominal CT images were extracted for all patients. Patients were included if they underwent CT within 3 weeks before the surgical operation. Patients were excluded if they were receiving dialysis therapy or had kidney morphological abnormalities such as cystic kidney or hydronephrosis. A history of coronary artery disease, heart failure, aortic aneurysm, or cerebrovascular disease was regarded as a macroangiopathy. Positive proteinuria was defined as proteinuria of 1+ or higher using an automated urine analyzer (US-2200; Eiken Chemical Co., Ltd., Tokyo, Japan). Urine albumin was not measured as a preoperative evaluation in this study. The body surface area was calculated by a square root method ^(12)^. After the chart review, 336 adult Japanese patients were eligible for analysis.

### Assessment of kidney parenchymal volume

**Figure 1.**
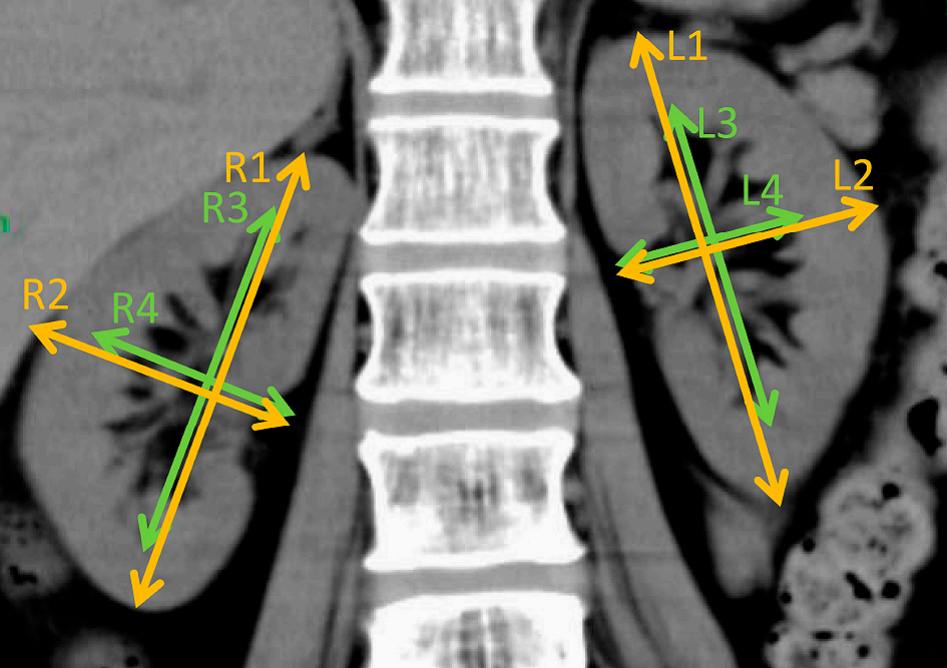
Coronal computed tomography image used to obtain total kidney volume. The maximum length of the kidney (R1-2, L1-2) and renal pelvis (R3-4, R3-4) was measured in the longitudinal and transverse axes. Using these lengths, the kidney volume was calculated by the equation described in the Methods section.

Kidney volume was measured by the ellipsoid method with modification using CT ^(13)^. Volume was calculated as the volume of a modified ellipse for each kidney and renal pelvis using the following formula: volume = π/6 × length × width^2^ in centimeters ^(14)^. Length and width were measured in centimeters using a coronal kidney image obtained by a 64-section helical CT scanner (Aquilion, Toshiba Medical Systems Corporation, Tochigi, Japan) in a 1-mm thick section (Figure 1). The length of the kidney or renal pelvis was measured parallel to the ipsilateral psoas major muscle. The width of the kidney or renal pelvis was also measured in centimeters, perpendicular to the ipsilateral psoas major muscle. Measurements of the kidney length were performed in duplicate by MM who was blinded to patients’ histories and laboratory results, and the mean length was used for further calculations. The parenchymal kidney volume was calculated by the subtraction of pelvic volume from kidney volume.

### Renal function evaluation

Serum creatinine was measured by an enzymatic method (TBA-c16000, Canon Medical Systems Co., Tochigi). In 255 patients, a urine collection was performed by self-urination or bladder catheter for 24 hour in a ward, and measured CCr (mCCr) was calculated as follows: 24-h CCr (mL/min) = {(urinary creatinine in mg/dL) × (24-h urine volume in mL)} / {(plasma creatinine in mg/dL) × 1440}. The estimated CCr (eCCr) was calculated using the Cockcroft–Gault equation ^(2)^: eCCr (mL/min) = {(140–(age in years))} x (weight in kg)} / {(serum creatinine in mg/dL) × 72} × (0.85 if female). The eGFR was calculated with the following Japanese equation ^(4)^: eGFR (mL/min/1.73 m^2^) = 194 × (serum creatinine in mg/dL) ^−1.094^ × (age in years) ^−0.287^ × (0.739 if female).

### Statistical analysis

Data was shown as mean ± standard deviation. Statistical comparisons were performed using an unpaired Student’s two-tailed t-test and Fisher’s exact test with Bonferroni correction or Pearson’s correlation analysis. Correlation coefficients and regression coefficients are shown as r and β, respectively. Multivariate regression analysis was performed by using all parameters shown in Table 2. The 95% confidence interval (CI) was also calculated. *P* < 0.05 was considered statistically significant. All statistical analyses were performed using EZR ^(15)^.

## Results

The characteristics of the patients in our study are shown in Table 1. The patients were 72.2 ± 13.2 years of age (Interquartile range: 67 to 82), 39.0% female, and all Japanese. The patients’ comorbidities included hypertension (54.8%), dyslipidemia (33.6%), and diabetes mellitus (26.5%). Patients with diabetes were treated with lifestyle modification (n = 44), oral antidiabetic agents (n = 43), or combination therapies with insulin (n = 2), resulting in adequate glycemic control (hemoglobin A1c 6.5 ± 0.9%). One hundred and fifty patients (44.6%) underwent surgery for malignant tumors; 72, 38, and 22 patients had colorectal, gastric, and liver cancer, respectively. A substantial number of patients (29.0%) had a history of macroangiopathies such as coronary artery disease or cerebrovascular disease, but any patient with amputation stump was not identified. The average mCCr, eCCr, and eGFR were 72.0 ± 21.5 mL/min, 64.7 ± 21.5 mL/min, and 68.6 ± 18.0 mL/min/1.73 m^2^, respectively. Statistically significant differences between female and male patients were found in the kidney volume, creatinine concentration, height, weight, and body surface area (Table 1). Interestingly, eCCr was significantly lower than mCCr, especially in female patients.

**Table 1.** Characteristics of Patients Undergoing Gastrointestinal Surgery in This Study.

	Unit	Total (336)	Female (131)	Male (205)
Patient parameters				
Age	year	72.2 ± 13.2	73.4 ± 13.7	71.6 ± 12.9
[interquartile range]		[67–82]	[66–83]	[67–82]
Height	cm	157.8 ± 9.4	149.9 ± 6.6^***^	162.9 ± 7.2
Weight	kg	55.2 ± 11.6	48.5 ± 9.0^***^	59.6 ± 10.9
BSA	m^2^	1.55 ± 0.19	1.42 ± 0.14	1.64 ± 0.17
Comorbidities				
Hypertension		54.8% (184)	50.4% (66)	57.6% (118)
Dyslipidemia		33.6% (113)	35.9% (47)	32.2% (66)
Diabetes mellitus		26.5% (89)	20.6% (27)	30.2% (62)
Macroangiopathy		29.0% (100)	29.0% (38)	30.2% (62)
Surgery				
Emergent		15.5% (284)	29.0% (29)	11.2% (23)
Malignancy		44.6% (150)	39.7% (52)	48.3% (99)
Renal parameters				
Right kidney volume	cm^3^	100.3 ± 27.6	91.6 ± 24.2^**^	105.9 ± 28.3
Left kidney volume	cm^3^	109.3 ± 30.9	100.9 ± 28.2^**^	114.7 ± 31.4
Creatinine	mg/dL	0.83 ± 0.30	0.71 ± 0.29^**^	0.90 ± 0.29
mCCr	mL/min	72.0 ± 21.5 (255)	71.1 ± 19.8 (91)	72.6 ± 22.4 (164)
eCCr	mL/min	64.7 ± 26.0##	58.6 ± 24.0^*^, ##	68.5 ± 26.5#
eGFR	mL/min/1.73 m^2^	68.6 ± 18.0	66.4 ± 16.6	70.0 ± 18.8
Proteinuria		12.5% (42)	11.5% (15)	13.2% (27)

Macroangiopathies included coronary artery disease, heart failure, cerebrovascular accident, and aortic aneurysm. The number of patients is indicated in parenthesis. The first and third age quartiles are shown within brackets. Other data are shown as mean ± standard deviation. ^*^p < 0.05, ^**^p < 0.01, and ^***^p < 0.001 compared with male patients by the two-tailed t test or Fisher’s exact test with Bonferroni correction. #p < 0.05, ##p < 0.01 compared with the eCCr by the paired t test with Bonferroni correction. BSA, body surface area; mCCr, measured creatinine clearance; eCCr estimated creatinine clearance; eGFR, estimated glomerular filtration rate.

**Figure 2.**
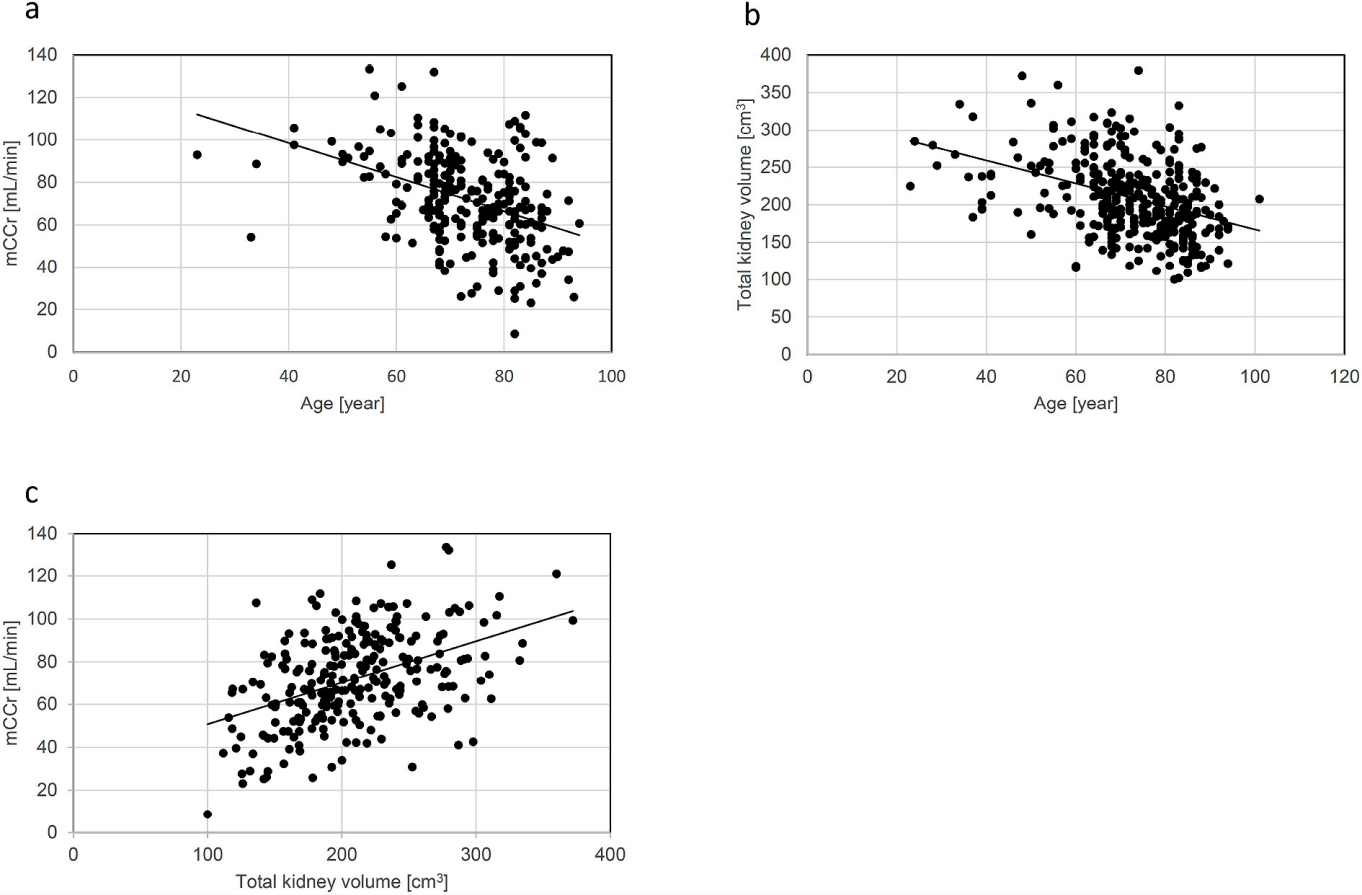
Correlations among age, mCCr, and total kidney volume. Scatter plots showed a significant negative correlation of age with (a) mCCr (r = −0.408, 95% CI = −0.506 to −0.300) and (b) total kidney volume (r = −0.387, 95% CI = −0.474 to −0.292). (c) The scatter plots showed a significant positive correlation of total kidney volume with mCCr (r = 0.445, 95% CI =0.341–0.538). mCCr, measured creatinine clearance; CI, confidence interval.

A scatter plot analysis was performed to investigate the relationship of age with kidney function or total kidney volume. As shown in Figure 2a, a negative correlation was found between age and mCCr (r = −0.408, 95% CI = −0.506 to −0.300, *p* < 0.01). In detail, the percentage of patients with mCCr of ≥ 60 mL/min was 81.3% among those aged 60 to 69 years, 71.1% among those aged 70 to 79 years, and 61.6% among those aged 80 to 89 years (data not shown). Age was also negatively correlated with the total kidney volume (r = −0.387, 95% CI = −0.474 to −0.292, *p* < 0.01) (Figure 2b). Next, we investigated the relationship between total kidney volume and mCCr. Notably, a significant positive correlation was found between them (r = 0.445, 95% CI = 0.341–0.538, *p* < 0.01) (Figure 2c) with a regression coefficient of 0.194 (95% CI = 0.146–0.243, *p* < 0.01).

A linear regression analysis was performed to determine factors associated with mCCr (Table 2). The univariate regression analysis showed age, height, hypertension, dyslipidemia, diabetes mellitus, macroangiopathy, proteinuria, malignant disease, and total kidney volume were significant parameters correlated with mCCr. After the multivariate regression analysis, age, diabetes mellitus, and total kidney volume remained statistically significant.

**Table 2.** Linear Regression Analysis for Predicting mCCr.

		Univariate		Multivariate	
		Regression coefficient (95%CI)	p value	Regression coefficient (95%CI)	p value
Age	(continuous)	−0.802 (−1.024 to −0.580)	< 0.001	−0.500 (−0.742 to −0.258)	< 0.001
Height	(continuous)	0.463 (0.174 to 0.752)	0.002	−0.144 (−0.562 to 0.274)	0.498
Weight	(continuous)	0.205 (−0.026 to 0.435)	0.081	0.008 (−0.277 to 0.293)	0.953
Gender	(female vs. male)	−1.508 (−7.042 to 4.026)	0.592	1.874 (−4.871 to 8.618)	0.585
Hypertension	(with vs. without)	−9.012 (−14.229 to −3.794)	< 0.001	−2.186 (−7.159 to 2.788)	0.388
Dyslipidemia	(with vs. without)	−6.718 (−12.179 to −1.258)	0.016	−2.561 (−7.697 to 2.576)	0.327
Diabetes mellitus	(with vs. without)	−9.915 (−15.816 to −4.014)	0.001	−8.184 (−13.794 to −2.573)	0.004
Macroangiopathy	(with vs. without)	−8.064 (−13.823 to −2.306)	0.006	−0.710 (−5.959 to 4.539)	0.790
Proteinuria	(with vs. without)	−12.999 (−23.511 to −2.487)	0.016	−1.523 (−11.218 to 8.167)	0.757
Total kidney volume	(continuous)	0.194 (0.146 to 0.243)	< 0.001	0.177 (0.120 to 0.233)	< 0.001

Univariate and multivariate linear regression analysis were performed using the parameters shown in Table 1. CI, confidence interval; mCCr, measured creatinine clearance.

**Figure 3.**
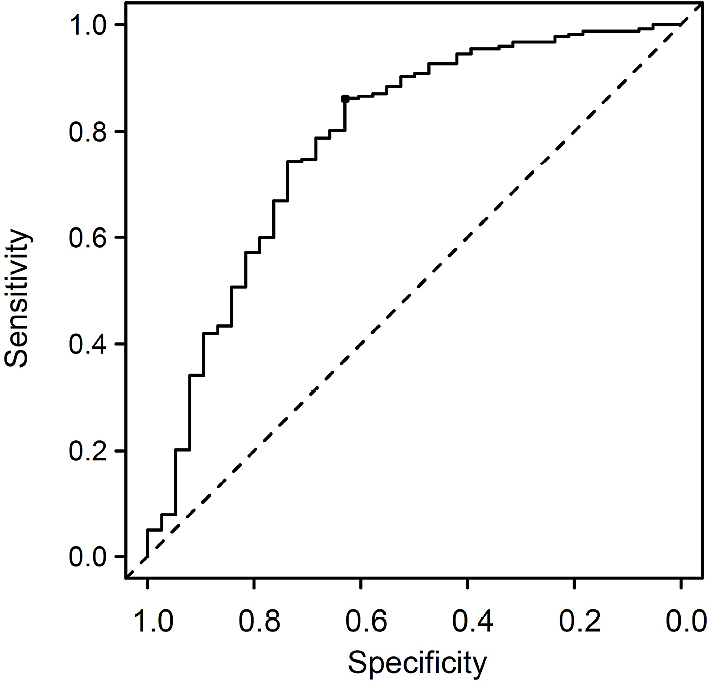
Receiver operating characteristic curves of total kidney volume to discriminate mCCr of ≥ 50 mL/min. The optimal cutoff value of the total kidney volume was 169.2 cm^3^ with a sensitivity of 86.2% and specificity of 63.2%. mCCr, measured creatinine clearance.

To challenge whether the total kidney volume as a univariable factor is useful for the prediction of mCCr in our cohort, a receiver operating characteristic curve analysis was performed to discriminate an mCCr of 30–60 mL/min in every 5 mL/min. As shown in Figure 3, use of the total kidney volume predicted mCCr of ≥ 50 mL/min with moderate accuracy (area under the curve = 0.782, 95% CI = 0.692–0.871), and the optimal cutoff level of the total kidney volume was 169.2 cm^3^ with a sensitivity of 63.2% and specificity of 86.2%.

## Discussion

In the present study, we analyzed preoperative laboratory data and CT images of patients who underwent gastrointestinal surgery and found a positive relationship between the total kidney volume and mCCr. Although there are several reports evaluating kidney volume and its function ^(6), (7), (8), (9)^, advantages of our study are (1) that we estimated the kidney volume by CT images, not ultrasonography; (2) that we used measured creatinine clearance, not estimated value; and (3) that we focused on the elderly, not the young. Although there is a similar study that showed a positive correlation between kidney volume calculated by CT images and measured CCr ^(16)^, these patients (58.1 ± 13.9 years) were significantly younger than those of our study (72.2 ± 13.2 years). Furthermore, mCCr was found to predict the total kidney volume obtained manually from CT images with moderate accuracy, even though age and the presence of diabetes mellitus were also found to be associated with renal function (Table 2) ^(1), (17)^. In the near future, the accuracy of predictive formulas may be improved by considering patient-related factors such as age and the diabetes mellitus status.

Many studies of kidney transplantation have revealed that split renal function is highly correlated with split renal volume ^(18), (19)^. This finding suggests that the kidney function per unit volume is constant within each individual. In contrast, the present study showed that the correlation between kidney volume and mCCr is present even in elderly individuals. As shown in Figure 2c, however, kidneys of almost the same volume showed a wide range in function. The density of the glomeruli declines with age, and the rate of decline is highly variable ^(20), (21)^. Therefore, the variation in the density of glomeruli might contribute to the wide range in function among kidneys of almost the same volume.

Performing CT frequently will not be accepted, because there is a potential risk of carcinogenesis by radiation exposure from abdominal CT scanning even in adults ^(22)^. The present study suggests that prediction of kidney function is reasonable by using pre-existing CT images. Since a previous study showed that dialysis-requiring acute kidney injury (AKI) following interventional coronary angiography did not develop in patients who had a baseline creatinine clearance of > 47 mL/min ^(23)^. Thus, prediction of creatinine clearance of ≥ 50 mL/min by pre-existing non-enhanced kidney images would provide useful information to avoid development of AKI induced by contrast media.

Measurement of the renal cortex volume seems to be a more reasonable methodology with which to assess kidney function than measurement of the parenchymal volume used in this study because the glomeruli are mainly located in the renal cortex ^(24), (25)^. However, evaluation of the cortex volume from CT images requires software with which to analyze the data and arterial-phase enhanced CT images, which should be avoided in patients with poor kidney function ^(26)^. A surveillance study of 1344 potential kidney donors showed that the median volume of the cortex, medulla, and parenchyma were 211, 80, and 292 mL, respectively ^(27)^, suggesting that the volumes of the renal parenchyma and cortex are generally consistent. Thus, evaluation of the renal parenchymal volume would provide a representative measurement of the cortex and serve as a simple unique method for assessing kidney function using noncontrast CT images.

Using magnetic resonance imaging (MRI) to estimate kidney volume seems also reasonable. The advantage of MRI is that the renal cortex and medulla are distinguishable without contrast media. A recent study using experimental chronic kidney disease model revealed that MRI parameters correlate with the renal histological feature ^(28)^. Although renal MRI scans are less frequently performed in clinical settings, MRI might provide more qualitative information of the kidney than noncontrast CT.

In 1976, Cockcroft and Gault developed a formula to predict CCr from 249 patients aged 18 to 92 years, and the formula requires multiplication by 0.85 for female individuals ^(2)^. However, a limitation of this widely accepted equation appears to be that only 59 patients aged ≥70 years participated in the study. As shown in Table 1, eCCr was significantly lower than mCCr, suggesting that eCCr predicted by the Cockcroft–Gault equation tends to be underestimated. This tendency was also reported by Ohara et al. ^(29)^, who surveyed 787 patients (80% male) with lung cancer at a median age of 70 years. Notably, the difference between mCCr and eCCr in the present study was greater in female patients (Table 1). Therefore, these observations raise the possibility that the Cockcroft–Gault equation is difficult to apply in the elderly population. However, further studies are needed to support this hypothesis.

The present study had several limitations. First, CCr was used to evaluate renal function. The GFR may be accurately measured by renal clearance of exogenous filtration markers such as inulin and other filtration markers ^(30)^. Nonetheless, such measurement is rarely performed in clinical practice because it is expensive and requires the use of radioelements for isotopic clearance determination. GFR which is estimated by cystatin-C was not assessed in this study, because these parameters were just estimated. Validation studies are needed to confirm the relationship between the total kidney volume and measured GFR. Second, we analyzed patients who underwent gastrointestinal surgery, 44.9% of whom had malignancy. Cachexia is often observed in patients with malignancy and affects the creatinine value, which in turn depends on the systemic muscle mass. Additionally, patients with cancer undergoing chemotherapy are often exposed to cytostatic agents that are potentially hazardous for renal function ^(31)^. Although the existence of a malignant tumor was not related to kidney volume in our multivariate regression analysis (Table 2), we cannot rule out the possibility that a decline in muscle mass affects the apparent creatinine value. Third, patients were excluded if they had kidney morphological abnormalities such as cystic kidney or hydronephrosis. Finally, this study was conducted in a single center, measurement of kidney length was performed by a single investigator, and included a relatively small number of patients. Further study is required to assess interinvestigator variability in measuring kidney length.

In summary, we found a positive relationship between total kidney volume, as assessed by the ellipsoid method, and kidney function and revealed the usefulness of kidney volume as a univariate predictor of renal function. The use of the total kidney volume predicted mCCr of ≥ 50 mL/min with moderate accuracy. These results might provide a unique method to predict development of AKI by using pre-existing kidney CT images.

## Article Information

### Conflicts of Interest

None

### Acknowledgement

We thank Dr. Shigeyo Tanabe, Dr. Kouji Matsuo and Dr. Masayuki Nakano for their helpful discussions. We also thank Angela Morben, DVM, ELS, from Edanz Group (https://www.edanzediting.com/ac) for editing a draft of this manuscript.

### Author Contributions

MM designed and performed the research; YF and HA supervised manuscript preparation.

### Approval by Institution Review Board (IRB)

This retrospective, cross-sectional study was approved by the Ethics Committee of Itoigawa General Hospital (No. 2017-9), and conducted in accordance with the principles of the Declaration of Helsinki.

### Informed Consent

Informed consent was not mandatory according to the ethical guidelines for epidemiological research in Japan.
